# Risk factors for cardiovascular morbidity among municipal public servants

**DOI:** 10.47626/1679-4435-2025-1407

**Published:** 2025-05-26

**Authors:** Giovanna Vallim Jorgetto, Fátima Livorato, Sandra Soares Mendes, Edna de Fátima Medeiros Neves, Jacqueline Bentitte Candido

**Affiliations:** Nurses. PhD Professors. University Center of the Associated Teaching Faculties (UNIFAE), São João da Boa Vista, SP, Brazil; Physician. MSc Professor. University Center of the Associated Teaching Faculties (UNIFAE), São João da Boa Vista, SP, Brazil; Nurse. Head of the Occupational Health Surveillance Sector, Municipal Government of São João da Boa Vista, São João da Boa Vista, SP, Brazil; Nurse. Coordinator of Primary Health Care, Municipal Government of São João da Boa Vista, São João da Boa Vista, SP, Brazil

**Keywords:** surveillance of the workers health, heart disease risk factors, cardiovascular diseases, epidemiology., vigilância em saúde do trabalhador, fatores de risco de doenças cardíacas, doenças cardiovasculares, epidemiologia.

## Abstract

**Introduction:**

Cardiovascular diseases are the leading cause of global mortality and significantly affect municipal public servants, particularly due to occupational stress and long working hours.

**Objectives:**

To assess cardiovascular risk factors diseases among public servants in a medium-sized municipality located in the eastern region of the state of São Paulo, Brazil.

**Methods:**

This was a cross-sectional study conducted in partnership with the Municipal Government of São João da Boa Vista, state of São Paulo, and the Centro Universitário das Faculdades Associadas de Ensino. The sample included 191 employees from general and specialized service sectors, with data collected in August 2022. The variables analyzed included demographic (age, sex, and educational attainment) and clinical data (physical inactivity, smoking, alcohol consumption, body mass index, waist circumference, and blood pressure). Statistical analyses were performed using tests with a significance level of 5% and 95%CI.

**Results:**

A higher prevalence of cardiovascular risk factors was observed among women, with statistically significant association for increased waist circumference (p < 0.001) and physical inactivity (p = 0.034). The main identified factors included obesity (40.8%), physical inactivity (68.0%), smoking (14.1%), and alcohol consumption (40.8%).

**Conclusions:**

Public servants showed multiple modifiable risk factors for cardiovascular diseases, notably obesity and physical inactivity. Recognizing these factors is crucial for designing health promotion interventions in the workplace.

## Abstract (pt)

**Introdução:**

As doenças cardiovasculares constituem a principal causa de mortalidade global e impactam de forma significativa os servidores públicos municipais, especialmente devido ao estresse ocupacional e às longas jornadas de trabalho.

**Objetivos:**

Avaliar os fatores de risco para doenças cardiovasculares em servidores públicos de um município de médio porte localizado no leste do estado de São Paulo.

**Métodos:**

Trata-se de um estudo transversal conduzido em parceria com a Prefeitura Municipal de São João da Boa Vista (São Paulo) e com o Centro Universitário das Faculdades Associadas de Ensino. A amostra foi composta por 191 servidores dos setores gerais e especializados, com dados coletados em agosto de 2022. As variáveis analisadas incluíram dados demográficos (idade, sexo e escolaridade) e clínicos (sedentarismo, tabagismo, consumo de álcool, índice de massa corporal, circunferência da cintura e pressão arterial). A análise utilizou testes estatísticos com nível de significância de 5% e intervalo de confiança de 95%.

**Resultados:**

Observou-se maior prevalência de fatores de risco cardiovascular em mulheres, com significância estatística para circunferência da cintura aumentada (p < 0,001) e sedentarismo (p = 0,034). Os principais fatores identificados foram obesidade (40,8%), sedentarismo (68,0%), tabagismo (14,1%) e consumo de álcool (40,8%).

**Conclusões:**

Os servidores apresentaram múltiplos fatores de risco modificáveis para doenças cardiovasculares, com destaque para obesidade e sedentarismo. O reconhecimento desses fatores é essencial para embasar intervenções voltadas à promoção da saúde no ambiente de trabalho.

## INTRODUCTION

Cardiovascular diseases (CVDs) are the leading cause of mortality worldwide.^1,2^ In Brazil, they account for approximately 30% of all deaths.^3^ Municipal public servants may be especially vulnerable to these diseases, due to work-related stress and long working hours.^4,5^ Occupational health is an extremely important topic for society, since workers’ health can directly affect productivity, quality of life, and safety at the workplace. Unfortunately, worker health care has proven insufficient in Brazil, representing a long-standing issue and a frequent target of criticism from several public health experts.^6^

Workers’ morbidity can have a significant impact on quality of life and productivity, and public servants are also affected by this problem.^7^ Therefore, it is important to study the risk factors for illness in municipal public servants, in order to prevent and treat diseases and promote better quality of life for these workers.^8,9^ The illness of municipal public servants is a matter of significant concern for both public health and public administration, as employee absences due to illness may hinder the functioning of public services and impose additional costs for local governments.^1,6,9^

Furthermore, the health of public servants is a matter of social justice, since these workers play a crucial role in delivering services to the population.^6,10,11^

The aim of this study was to evaluate CVD risk factors among public servants in a medium-sized municipality in the eastern region of the state of São Paulo, Brazil.

## METHODS

A cross-sectional study was conducted in partnership with the Municipality of São João da Boa Vista, state of São Paulo, Brazil, and the Centro Universitário das Faculdades Associadas de Ensino (UNIFAE) of São João da Boa Vista, state of São Paulo, Brazil.

The target population consisted of 268 servants allocated in the Kitchen and General/Specialized Services sectors. Inclusion criteria were being a statutory public servant at the municipality of São João da Boa Vista, state of São Paulo, Brazil, being over 18 years of age, agreeing to participate in the project, and having the capacity to understand the Informed Consent Form. Temporary servants, those employed under the Consolidation of Labor Laws (Consolidação das Leis do Trabalho, CLT) regimen, interns, and individuals younger than 18 years of age were excluded.

The final sample consisted of 191 servants, due to absenteeism of 77 servants (28.73%). Data were collected from August 20 to 27, 2023, at previously scheduled times.

A demographic description of the sample was provided, including data on age, sex, and educational attainment. Study variables included CVD risk factors, physical inactivity, smoking, alcohol consumption, blood pressure (BP), waist circumference (WC), hip circumference (HC), and body mass index (BMI).

Body mass was measured using a Plenna digital scale with a 5 x 2.5 cm liquid crystal display, powered by a lithium battery, with 100 g precision and a maximum capacity of 150 kg. All individuals were instructed to wear light clothing, remove their shoes, and stand upright with feet placed side-by-side and arms extended to the sides.

Height data were obtained by self-report, because the questionnaires were administered at the servants’ workplace. Epidemiological studies monitoring overweight prevalence in the population have shown that self-reported height is a reliable source of information.^12^

These measures were then used to calculate BMI, by dividing weight (kg) by the square of height (m). The results were classified according to cutoff points recommended by the World Health Organization, so that participants whose BMI was from 18.5-24.9 kg/m^2^ were classified as normal weight, those with BMI from 25.0-29.9 kg/m^2^ were classified as overweight, and those with BMI ≥ 30.0 kg/m^2^ were classified as obese.^13^ Similar to the other independent variables of the present study, BMI was also dichotomized, i.e., reclassified as normal weight or overweight/obesity.

WC was measured using a non-extensible fiberglass measuring tape (Cardiomed brand), 200 cm in length, with a precision of 0.1 cm. The measurement was taken at the midpoint between the iliac crest and the last rib, with interpretation based on the guidelines established by National Institutes of Health.^14^ For men, a WC of < 94.0 was considered “low risk” and ≥ 94.0 cm “moderate/high risk”; for women, values < 80.0 cm indicated “low risk”, and values ≥ 80.0 cm indicated “moderate/high risk”.

HC was measured by running non-extensible fiberglass measuring tape around the hips at the point of greatest protuberance, without compressing the skin. The results for WC and HC were used to calculate the waist-to-hip ratio (WHR), for which the cutoff points adopted for “normality” were up to 0.95 cm for men and up to 0.85 cm for women.

Finally, BP readings were taken using an OMRON automatic BP monitor, model HEM-742INT, with a digital display and a measurement range of 0 to 299 mmHg. The measuring procedures and classification of results were conducted in accordance with American Heart Association recommendations.^15^ BP readings were classified using the following cutoff points: i) systolic BP (SBP) ≤ 120 mmHg and diastolic BP (DBP) ≤ 80 mmHg for “ideal”; and SBP > 120 mmHg and DBP > 80 mmHg for “not ideal.” Additionally, individuals who stated that they had hypertension and were regularly taking antihypertensive medication were also classified as having “not ideal” BP. If a participant’s SBP and DBP were in different categories, they were classified according to the higher of the two, i.e., as “not ideal.”

The study was approved by the Research Ethics Committee under Certificate of Presentation for Ethical Approval (Certificado de Apresentação para Aprovação Ética, CAAE) number 56912722.0.0000.5382 and complied with the ethical standards of Resolution number 466/2012 of the Brazilian National Health Council.

Statistical analyses were performed using the software Stata 12.0 (StataCorp, College Station, USA). Data were assessed using statistical tests, including chi-square test and prevalence ratio (PR), considering a significance level of 5% and a 95%CI.

## RESULTS

Of the 191 study participants, 76 were men and 115 were women. Mean age was 45 years, ranging from 24 to 68 years in both sexes, with a predominance of workers aged from 40 to 49 years (37%). With regard to educational attainment, 79% of women and 76% of men had more than 8 years of education (Table 1).

**Table 1:** Demographic profile according to sex, age, and educational attainment among municipal public servants in the state of São Paulo, 2024

Variables	Total		Women		Men		p-value^*^
	n	%	n	%	n	%	
Age (years)							
≤ 39	57	29.8	30	15.7	27	14.1	0.163
≥ 40	134	70.1	85	44.5	49	25.6	
Educational attainment (years)							
≤ 8	38	19.8	21	10.9	17	8.9	0.514
> 8	149	78.0	91	47.6	58	30.3	

* p-value obtained using the chi-square test.

Source: The authors (2025)

Cardiovascular risk factors according to sex are displayed in Table 2, revealing a higher prevalence of cardiovascular risk among women compared to men, with the exception of smoking and alcohol consumption. Significant associations were observed for increased WC and physical inactivity. Table 3, in turn, describes the prevalence of CVD risk factors, also according to sex.

**Table 2:** Cardiovascular risk factors according to sex among municipal public servants in the state of São Paulo, 2024

Risk factors	Total		Women		Men		p-value^*^
	n	%	n	%	n	%	
BMI							
Normal weight	38	19.8	21	10.9	17	8.9	0.186
Overweight	70	36.6	38	19.8	32	16.7	
Obesity	78	40.8	53	27.7	25	13.0	
WC							
Increased	73	38.2	48	25.1	25	13.0	< 0.000^*^
Normal	80	41.8	29	15.1	51	26.7	
WHR							
Increased	78	40.8	53	27.7	25	13.0	0.213
Normal	113	59.1	86	45.0	27	14.1	
Clinical examination							
High SBP	108	56.5	64	33.5	44	23.0	0.265
High DBP	69	36.1	35	18.3	34	17.8	
Life habits							
Physical inactivity	130	68.0	80	41.8	50	26.1	0.034^*^
Alcohol consumption	78	40.8	34	17.8	44	23.0	
Smoking	27	14.1	13	6.8	14	7.3	

WC = waist circumference; BMI = body mass index; DBP = diastolic blood pressure; SBP = systolic blood pressure; WHR = waist-to-hip ratio.

* Statistically significant value

** p-value obtained using the chi-square test.

Source: The authors (2025)

**Table 3:** Prevalence and prevalence ratio of cardiovascular diseases risk factors, according to sex, among municipal public servants in the state of São Paulo, 2024

Variables	Total	Women		Men		PR	Adjusted
	%	n	%	n	%	Crude	
BMI	77.0	91	79.0	57	75.0	1.93	1.87
High WHR	75.0	88	78.0	70	70.0	2.04	2.01
High SBP	57.0	64	56.0	44	58.0	1.41	1.41
High DBP	36.0	35	30.0	34	43.0	1.86	1.86
Physical inactivity	68.0	80	70.0	50	66.0	1.99	1.96
Smoking	14.0	13	11.0	14	18.0	1.72	1.72
Alcohol consumption	41.0	34	30.0	44	58.0	1.92	1.85
Accumulated risk							
One	50.0	51	26.7	29	15.1	0.58	0.58
Two	37.0	22	11.5	26	13.6	1.26	1.36
Three or more	22.0	28	14.6	45	23.5	1.97	1.97

BMI = body mass index; DBP = diastolic blood pressure; SBP = systolic blood pressure; WHR = waist-to-hip ratio; PR = prevalence ratio.

Source: The authors (2025)

## DISCUSSION

The illness process among workers has received considerable attention from researchers in recent years. However, despite the extensive body of literature on the topic, important gaps remain, such as promoting the effective participation of workers in managing their own health and work processes.^16^

Therefore, the identification and monitoring of cardiovascular risk factors associated with worker illness is crucial, considering their growing link to potentially detrimental health effects, negative impacts on quality of life, and their emergence as a current public health issue.^17^

Various risk factors are associated with the development of CVDs, and these can be classified as modifiable and non-modifiable. Modifiable risk factors include hyperlipidemia, smoking, alcohol consumption, hyperglycemia, obesity, physical inactivity, poor diet, and use of contraceptives. These factors were identified and/or altered in our study, with the exception of contraceptive use, which was not addressed in our survey. Non-modifiable risk factors include family history of CVD, age, sex, and race.^18^ In the present study, the majority of participants were women, mostly over the age of 40 and of white skin color, which contrasts with literature indicating that CVD is more prevalent among men than women.^19^

This study analyzed the profile of 191 municipal public servants who participated in a survey, which indicated a higher prevalence of cardiovascular risk factors among women, particularly increased BMI, WHR, and BP. This may suggest that women are more aware of their actual health status, which resulted in data more consistent with of adiposity and BP indicators identified.

Excess weight is associated with the development of physiological and metabolic disorders such as arterial hypertension, lipid profile alterations, and hyperinsulinemia. These conditions, which are part of metabolic syndrome, are also related to the onset of CVDs and type 2 diabetes mellitus.^20^ The present study corroborates these findings, with 10% of participants diagnosed with diabetes, 32% with arterial hypertension, and between 13 and 15% exhibiting lipid abnormalities.

The persistence of excess body weight is associated with increased abdominal circumference and therefore with a higher WHR, both of which are commonly employed as diagnostic criteria for CVD risk assessment. A study involving female employees at a public school found that 38.5% exhibited increased abdominal circumference.^20^ Another study conducted with civil police officers in the Brazil’s Federal District found a higher prevalence of increased abdominal circumference among women (68%), compared to men (45%).^21^

The participants in this study showed BP levels above the reference values, which were associated with weight gain, consistent with previous research.^22^ These findings are also in agreement with previous studies that demonstrated an association between systemic arterial hypertension (SAH) and overweight/obesity in both men and women. Another study reported a 36.8% prevalence of SAH among obese individuals, representing a PR 5.08 times higher compared to normal weight individuals.^23^ The characteristics of individuals presenting hypertensive peaks are cause for concern, considering that weight gain promotes physiological mechanisms resulting in hyperinsulinemia, which at the renal level induces vasoconstriction and hypertension.^24^_._

The limitations of the study include the fact that the sample was limited to servants from a single municipality, restricting the generalization of the findings, and the cross-sectional design, which does not does not allow for causal inferences. Longitudinal studies are recommended to further investigate these findings.

## CONCLUSIONS

Hyperlipidemia, smoking, alcohol consumption, obesity, and physical inactivity were identified as modifiable cardiovascular risk factors among the workers evaluated in this study.

Women showed a higher prevalence of cardiovascular factors, such as increased BMI, WHR, and BP.

Recognizing workers’ risk factor profiles is essential for guiding health promotion measures, particularly in light of the findings of this study and the high cardiovascular morbidity and mortality rates in developing countries.
